# Matrixing Designs for Shelf-Life Determination of Parenteral Drug Product: A Comparative Analysis of Full and Reduced Stability Testing Design

**DOI:** 10.3390/pharmaceutics16091117

**Published:** 2024-08-24

**Authors:** Lara Pavčnik, Igor Locatelli, Tina Trdan Lušin, Robert Roškar

**Affiliations:** 1 SANDOZ Development Center Slovenia, Lek Pharmaceuticals d.d., Verovškova 57, 1526 Ljubljana, Slovenia; lara.pavcnik@sandoz.com (L.P.); tina.trdan_lusin@sandoz.com (T.T.L.); 2 Department of Biopharmaceutics and Pharmacokinetics, Faculty of Pharmacy, University of Ljubljana, 1000 Ljubljana, Slovenia; igor.locatelli@ffa.uni-lj.si

**Keywords:** shelf-life, matrixing design, ICH Q1D, stability study, degradation kinetics, parenteral dosage forms

## Abstract

This article highlights the applicability of matrixing designs in stability studies for parenteral medications. The traditional approach involves extensive testing over the product’s shelf-life. However, matrixing designs offer an alternative approach where only a fraction of samples is tested at each time point. The study conducted in this article focused on three parenteral medications and examined stability data under long-term condition. Degradation products were identified as critical parameter, and kinetics of degradation varied among the selected products. A systematic methodology was adopted to evaluate the data using different matrixing designs. The regression models obtained were assessed using statistical parameters S and R^2^. Also, each of the 28 matrixing designs were compared to the full design with statistical parameter RMSE and the shelf-life. The results confirmed that each of the evaluated matrixing designs can be applied, whether degradation product shows a linear or non-linear increase, and demonstrated that a reduction of two time points per batch is the most appropriate. In conclusion, this research contributes to the understanding of utilizing reduced matrixing designs in stability studies for parenteral medications and can be an effective strategy to reduce costs and time of stability testing while maintaining the necessary level of precision and reliability.

## 1. Introduction

Parenteral preparations are sterile preparations intended for administration by injection, infusion or implantation into the human or animal body. Parenteral administration offers several advantages over other routes of administration. These advantages include rapid action, reduction in hepatic metabolism, elimination of gastrointestinal inactivation, the possibility of administration when oral administration is not possible (e.g., when the patient is unconscious), easier access to certain compartments (e.g., articulation), and direct administration of nutrition [1]. Since parenteral products are introduced by bypassing the body’s most important protective barriers, the skin and mucous membranes, they must be “essentially” free of physical, chemical, and biological impurities. Parenterally administered drugs are relatively unstable and generally highly potent drugs that require strict control of administration to the patient. Overcoming stability issues and achieving and maintaining sterility and other purity requirements are major challenges for those who develop, manufacture and administer sterile drug products [2,3].

According to the International Council for Harmonization (ICH) guideline Q1A (R2), the purpose of stability testing is to provide evidence on how the quality of a drug substance or product varies with time under the influence of a variety of environmental factors, such as temperature, humidity, and light. In addition, all dosage forms must be stable under predetermined manufacturing, packaging, and usage conditions. The aim of the stability study is, therefore, to assess the changes during the study and to establish the shelf-life and recommended storage conditions for the drug product [4]. The stability of the active pharmaceutical ingredient is a key factor in the effectiveness of a parenteral drug product. A drug product must maintain potency relative to label claim over the shelf-life to deliver an accurate dose [5]. Stability studies should include testing of those attributes of the drug product that are susceptible to change during storage and are likely to influence quality, safety, and efficacy [4]. Parenteral dosage forms, like all other dosage forms, need to maintain both chemical and physical stability throughout the shelf-life of the product. Moreover, sterility evaluation is an additional stability requirement for sterile dosage forms. Therefore, product stability includes not only chemical and physical properties, but also microbiological stability (i.e., maintenance of sterility and apirogenicity) throughout the shelf-life [3].

The traditional design of a stability study for parenteral dosage forms is based on a large number of experiments. ICH Q1A (R2) recommends that at least three batches of a drug product of all strengths and filling volumes intended for the market must be tested under prescribed storage conditions and time points. Furthermore, different orientations, upright and inverted or horizontal, need to be evaluated. In general, the long-term stability of products should be tested every three months in the first year, every six months in the second year, and once a year throughout the proposed shelf-life. Long-term conditions should correspond to the storage conditions indicated on the container label (e.g., 25 °C ± 2 °C/60% RH ± 5% RH or 30 °C ± 2 °C/65% RH ± 5% RH). Drug products should also be stored under accelerated conditions, that is at 40 °C ± 2 °C/75% RH ± 5% RH for six months, tested at least at zero, three, and six months in case of drug products with storage conditions at room temperature. Furthermore, when long-term studies are conducted at 25 °C ± 2 °C/60% RH ± 5% RH and significant change occurs at any time point during six months of testing under accelerated conditions, additional testing at an intermediate storage condition should be performed [4,6]. Such an amount of stability experiments leads to a very extensive and expensive study design.

As an alternative, ICH Q1D (Bracketing and Matrixing Designs for Stability Testing of New Drug Substances and Products) allows the use of reduced designs in stability studies. The matrixing design is defined as a statistical design of a stability schedule so that only a fraction of the total number of samples is tested at a given sample time point. The design assumes that the stability of each subset of samples tested represents the stability of all samples at a given time point [7].

Reduced stability studies have been evaluated in the past, but there are few data available. Furthermore, no articles on this topic have been published recently. Considering reduced experimental designs, Nordbrock suggested that the best design is the one with the highest power among all designs with a fixed sample size, or the design with the smallest sample size among those that have at least the desired power [8]. Instead of Nordbrock’s approach, Chow suggested comparing the precision of the estimated expiration dating [9]. Furthermore, Natarajan et al. discussed the effects of pharmaceutical compounds on the expiration dating period of various matrixing designs on the choice of sampling times [10]. DeWoody and Raghavarao also evaluated some optimal matrixing designs in stability studies by choosing the time vectors such that the design is optimal in terms of maximum information per unit cost [11]. Oliva et al. evaluated if pooling data for several batches were feasible using the matrix and full-testing approaches to determine a common shelf-life [12]. Reduced stability testing is considered by pharmaceutical companies to be very suitable for reducing the costs of stability studies. However, in the articles mentioned above, the matrixing testing was mainly evaluated for solid pharmaceutical dosage forms.

Our aim was to investigate the feasibility of implementing reduced matrixing designs in parenteral dosage forms. Although reduced stability studies are permitted in line with ICH Q1D, industrial companies have been cautious about using this approach due to the lack of literature data for studies conducted on parenteral products. To evaluate the suitability of reduced matrixing designs, three parenteral dosage forms were selected, and their stability data under long-term conditions were investigated. In all three cases, critical attributes were selected for determining the proposed shelf-life. In addition, the nature of the degradation relationship was examined to determine whether the data exhibited linear or non-linear (e.g., quadratic polynomial) patterns over the shelf-life. A systematic methodology consistent with ICH guidelines was used to evaluate the stability information [3,4]. First, the least stable filling volume was selected for each product. Subsequently, an evaluation of the batch-to-batch variability was carried out. Finally, the long-term stability data were analyzed using different matrixing designs, and the resulting regression models were assessed using statistical parameters.

## 2. Materials and Methods

### 2.1. Products

Three parenteral pharmaceutical products developed and manufactured in Sandoz were evaluated. Pemetrexed is available as a solution for infusion into a vein; 1 mL of the product contains pemetrexed sodium equivalent to 25 mg pemetrexed. It was produced in three filling volumes, 100 mg/4 mL, 500 mg/20 mL, and 1000 mg/40 mL, and packed in clear, colorless type I glass vial with a bromobutyl rubber stopper and aluminum crimp cap with light blue plastic flip-off. Sugammadex is a solution for injection administered intravenously as a single bolus injection. A 1 mL of the product contains sugammadex sodium equivalent to 100 mg sugammadex. It was produced in two filling volumes, 200 mg/2 mL and 500 mg/5 mL, and packed in type I glass vial closed with bromobutyl rubber stopper with an aluminum cap and flip-off seal. Docetaxel is available as a solution for infusion into a vein. A 1 mL of the product contains docetaxel trihydrate equivalent to 10 mg docetaxel. It was produced in three filling volumes, 20 mg/2 mL, 80 mg/8 mL, and 160 mg/16 mL, and packed in a type I glass vial with a rubber stopper (latex free) and flip-off aluminum crimp. Structures of the examined compounds are presented in the Supplementary Materials (Figure S1).

### 2.2. Stability Study Design

For each pharmaceutical product and each filling volume, three different batches were produced and stored in stability chambers under different storage conditions. The long-term stability study was performed under conditions of 25 °C ± 2 °C/60% RH ± 5% RH. Following ICH Q1A (R2), the pull points of the stability study were 3, 6, 9, 12, 18 and 24 months. In addition, all batches were subjected to an accelerated stability study at 40 °C ± 2 °C/75% RH ± 5% RH for six months, tested at 0, 3, and 6 months and an intermediate stability study at 30 °C ± 2 °C/75% RH ± 5% RH for 12 months, tested at 0, 6, 9 and 12 months [4,6]. Stability studies were conducted in all conditions in two orientations: upright and inverted/horizontal orientation. The design of the stability study is summarized in Table 1.

### 2.3. Tested Parameters and Analytical Methods

Stability studies should include testing of those attributes of the drug product that are susceptible to change during storage—stability-indicating parameters. Parenteral dosage forms need to maintain chemical (assay, degradation products), physical (appearance, color, pH, osmolality, visible particles, subvisible particles), and microbiological (sterility, bacterial endotoxins) stability throughout the shelf-life of the product [3,4]. For all three parenteral drug products evaluated in this article, all of the above listed parameters were analyzed. Quantitative parameters, i.e., assay, pH, and osmolality, remained within the specified limits (specified limit for assay is 90.0–105.0% for Pemetrexed, Sugammadex and Docetaxel; the specified limits for pH are: 7.0–9.0 for Pemetrexed, 7.0–8.0 for Sugammadex, and 3.0 to 4.5 for Docetaxel, and the specified limit for osmolality is 300–500 mOsmol/kg for Sugammadex).

During the shelf-life, an increasing trend in oxidative degradation products was observed, and therefore, the statistical analysis was performed for the mentioned parameter. For the parenteral drug product Pemetrexed, the oxidative degradation product pemetrexed S-dimer (according to USP); for Sugammadex, mono-S-oxo-sugammadex (chemical name); and for Docetaxel, 6-oxodocetaxel (according to USP) were investigated. All three degradation products are referred to as IMP1 in this article. Structures of monitored degradation products are presented in the Supplementary Materials (Figure S2). Validated high-performance liquid chromatography (HPLC) and ultra-performance liquid chromatography (UHPLC) methods were used to determine the degradation products. The chromatographic conditions for the analytical methods used are listed in the Supplementary Materials (Table S1).

### 2.4. Matrixing Designs

To evaluate the long-term stability data of the parenteral pharmaceutical products, several matrixing designs were tested, involving all possible combinations of the time point selections (Table 2). Additionally, the time points (months) of 0, 12, and 24 were fixed and the variation among the batches was not included. The same matrixing design combinations were applied in the study of DeWoody and Raghavarao [11].

### 2.5. Data Analysis

A systematic approach was adopted based on ICH guidelines in the presentation and evaluation of the stability information [3,4]. First, the long-term stability data for all filling volumes and attributes were statistically evaluated using regression analysis to determine the less stable factor combination. As three different batches were produced for each product and filling volume, an analysis of covariance (ANCOVA) was performed to assess the batch-to-batch variability. If the analysis showed that the batch-to-batch variability is low, the data could be combined into an overall estimate. For this purpose, appropriate statistical tests (e.g., *p*-values for a significance level of rejection of more than 0.25) were applied to the slopes of the regression lines and the zero-time intercepts for the individual batches [3,13]. The data analysis was performed using Minitab^®^ 20.3 (Minitab Inc., State College, PA, USA).

After an evaluation of less stable factor combination (filling volume and testing parameter) and batch-to-batch variability, the long-term stability data were evaluated by different matrixing designs. Furthermore, since the kinetics of the increase in degradation products can be linear or non-linear, the matrixing designs for different degradation kinetics were evaluated. In the case of non-linear kinetics, a quadratic regression model was applied. The obtained regression models were evaluated with statistical parameters such as standard error of regression (S) and coefficient of determination (R^2^). Higher R^2^ and lower S values indicate a stronger fit of the data to the regression model. The regression models were built using with Minitab^®^ 20.3 (Minitab Inc., US). The shelf-life was also estimated using Minitab and considering the specification limits for each product. The calculations of shelf-life for the comparison of matrixing designs were performed without considering the 95 one-sided confidence limit.

In addition, the accuracy of the 28 matrixing designs (M2 to M29; Mj) in comparison to the full design (M1) was evaluated by root-mean-square error (RMSE). RMSE was calculated for each matrix design (Mj) using 6 (non-zero) time points (i) and predicted values from the regression (y-hat):

$${RMSE}_{M_{j}\;vs\;M1}=\sqrt{\frac{1}{6}\times \sum_{i=1}^{6}{\left({\hat{y}}_{i}^{M_{j}}-{\hat{y}}_{i}^{M1}\right)}^{2}}$$

We also established a threshold for our data, determining that an RMSE of 0.01% or lower is considered acceptable. This threshold was set by considering the quantification limit of the analytical method for the degradation products, which was 0.05% (reporting threshold for impurities [14]) in all three cases, as well as the set permitted method error of 20% at this low concentration level (0.05%).

## 3. Results

### 3.1. Evaluation of the Least Stable Filling Volume and Batch-to-Batch Variability

The stability data showed slight variability in the quantitative parameters, i.e., assay (results from 96.1% to 101.7% for Pemetrexed, results from 98.1% to 101.0% for Sugam-madex, and results from 95.1% to 99.2% for Docetaxel), pH (results from 7.6 to 8.2 for Pemetrexed, results from 7.6 to 7.9 for Sugammadex, and results from 3.3 to 3.4 for Docet-axel), and osmolality (results from 321 mOsmol/kg to 342 mOsmol/kg for Sugammadex), but no trends were observed, and all results remained within the specified limits. During the shelf-life, an increasing trend in oxidative degradation products was observed, and therefore, the statistical analysis was performed for this parameter.

Degradation product data of a completed long-term stability study for all three products manufactured in different filling volumes are presented in Tables S2–S4. Statistical evaluation was performed for filling volumes of 100 mg/4 mL for Pemetrexed, 200 mg/2 mL for Sugammadex, and 20 mg/2 mL for Docetaxel, since a higher increase in main degradation product IMP1 during shelf-life was noticed.

An analysis of covariance (ANCOVA) was performed from the results in Table S5 to evaluate the variability between the individual batches, as three batches were produced for each filling volume. The performed analysis of *p*-values for slopes (batch*time) and intercepts of regression lines (batch) obtained with the ANCOVA model for all three products show that the batch-to-batch variability was higher than 0.25; therefore the data could be combined into an overall estimate.

### 3.2. Regression Analysis

**Figure 1:**
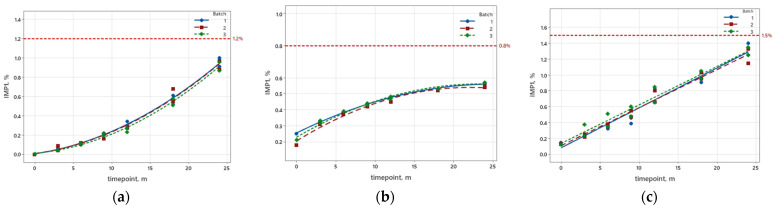
Increase in degradation products during the shelf-life for three batches. The red line represents the specification limits for oxidation-related degradation products, which were (**a**) 1.2% for Pemetrexed, (**b**) 0.8% for Sugammadex, and (**c**) 1.5% for Docetaxel.

According to ICH Q1E, we developed a regression model for each product and used this model to predict the shelf-life. As shown in Figure 1, the increase in oxidative degradation products in the completed long-term stability study was non-linear for the products Pemetrexed and Sugammadex, but linear for Docetaxel.

Despite the nature of the degradation kinetics, Pemetrexed and Sugammadex were also evaluated with non-linear regression analysis, while for Docetaxel, only a linear regression analysis was performed for all 29 designs, as shown in Table 3. The adequacy of the regression models obtained was evaluated using the statistical parameters standard error of regression (S) and coefficient of determination (R^2^). The RMSE was also calculated to identify the matrixing design (M2–M29) that was most comparable to the full design M1. Evaluated data are presented in Tables S6–S8.

**Figure 2:**
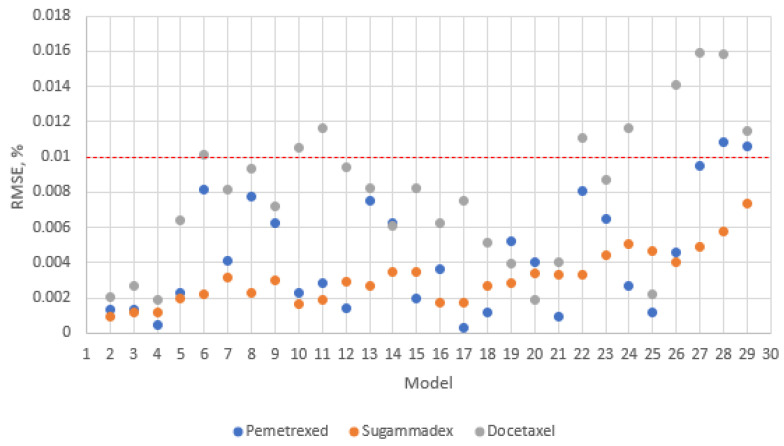
Graphical presentation of RMSE for reduced models obtained with non-linear regression for Pemetrexed and Sugammadex and with linear regression for Docetaxel. The red line represents the RMSE threshold (0.01%).

The statistical parameter RMSE for all 28 reduced designs obtained with the corresponding regression model is shown graphically in Figure 2.

### 3.3. Shelf-Life Calculation

To test the influence of the regression model on the calculated shelf-life, the data were analyzed using linear and non-linear regression. For all three products, a linear regression model was used to predict the shelf-life with data for 12 months and 24 months (Table S9). The data for Pemetrexed, for which the increase in oxidative degradation products from the completed long-term stability study was non-linear, were also analyzed using a non-linear regression model to predict shelf-life using 12- and 24-month data. Summarized results of statistical analysis are shown in Table 4.

## 4. Discussion

The quantitative attributes of all three parenteral drug products were assessed, and it was found that degradation products are the critical attribute that increases with time. Since the three parenteral drug products considered were manufactured in varied filling volumes, an assessment was performed to determine the filling volume with the lowest stability. The less stable filling volume was found to be 100 mg/4 mL for Pemetrexed, 200 mg/2 mL for Sugammadex, and 20 mg/2 mL for Docetaxel, which is evident from stability data (Tables S2–S4). For Pemetrexed, a significant increase in impurities was observed in the lowest filling volume compared to the higher filling volumes. In contrast, the differences in impurities between the filling volumes for Sugammadex and Docetaxel were smaller, but the trend was the same, less stable at the lower filling volumes.

It is important to note that the drug substances in all three tested products were subjected to oxidation, as in each case, an oxidative degradation product was the critical quality attribute. Based on additional experiments, it was found that the formulation with the highest headspace to filling volume ratio was most affected by the increase in degradation products. In addition, during the stability studies, the oxygen content in the headspace of the vial increased due to minimal gas exchange through the container closure. The amount of oxygen entering the vial through the container closure was found to be an important factor influencing the stability of the drug product. The increase in oxygen depended on the combination of the vial neck/closure and the volume of the headspace. The formulation with the smallest filling volume was most affected by oxygen permeation and subsequent oxidation of the formulation, which is why this worst-case scenario was selected for each tested drug product for further work.

For statistical analysis, it is advantageous to combine data from different batches into an overall estimate, as this brings (i) higher accuracy, leading to a more precise and accurate prediction of shelf-life and reducing the likelihood of over- or underestimation of product stability; and (ii) higher reliability, allowing for a more robust assessment as it reduces the influence of individual data points or outliers [15]. Analysis of covariance (ANCOVA) showed that the batch-to-batch variability is small; therefore, the data were combined into one overall estimate. Namely, *p*-values below the ICH significance level of 0.25 indicate that the F-test for the separate slopes was significant, and considerable batch-to-batch variability could be confirmed [16].

### 4.1. Regression Analysis

Typically, a linear relationship between certain quantitative attributes and time is assumed [17]. However, according to ICH guideline Q1A(R2), the nature of the degradation relationship determines whether data should be analyzed using linear or non-linear regression [4].

As shown in Figure 1, the increase in degradation products for the product Pemetrexed was not linear. Initially, the increase was slower, but after 12 months, the increase was exponential. For this set of stability data, a linear and non-linear regression analysis was performed for all 29 designs, as shown in Table 3. Since M1 is the full design, we consider that the results obtained are the most correct and accurate for the statistical parameters (R^2^ and S). When analyzing data with non-linear regression, the obtained R^2^ was higher and S is lower for all designs, which means that the model fit the data well. This was to be expected, as the increase in degradation products was not linear. By using linear regression in this case, we made a bigger error in the statistical analysis. In addition, the values for the reduced models (M2–M29) were comparable to those of the full design, so we can conclude that all regression models fit the data in a similar way. Furthermore, the shelf-life was calculated for all reduced models with linear and non-linear regression. The results show that the calculated shelf-life with linear regression was higher than that calculated with non-linear regression for all designs. Since the nature of the increase in degradation products was not linear, we obtained a more accurate prediction of shelf-life when using non-linear regression.

In contrast to Pemetrexed, the increase in the degradation product in Sugammadex was initially faster and slowed down after 12 months. However, the increase in the degradation product was still non-linear. As with Pemetrexed, the parameter R^2^ was also higher for Sugammadex, and S was lower for all matrixing designs analyzed with non-linear regression (Table 3). The shelf-life, on the other hand, could only be calculated for linear regression. In this case, when we consider the non-linear regression, the curve did not exceed the specification limit. For the product Docetaxel, the stability data were only analyzed with linear regression since the increase in the degradation product was linear (Figure 1). From the results of the statistical parameters (Table 3), we can notice that S, R^2^, and shelf-life were similar for all matrixing designs. However, RMSE slightly increased when reducing the time points.

From the obtained results of the statistical parameters R^2^ and S, it can be concluded that, depending on the degradation kinetics, the correct regression analysis, linear or non-linear, must be used to evaluate the stability data. However, the values between the reduced models determined in the same regression analysis did not differ notably. This indicates that all reduced models effectively fit the stability data. However, when comparing the results of the same product obtained with linear regression and non-linear regression, there were clear differences. It is crucial to use the appropriate regression method for data evaluation, as this helps to minimize the errors in predicting shelf-life.

### 4.2. Shelf-Life Determination

According to ICH Q1A (R2), the initial application to regulatory agencies for a drug product should include at least 12 months of stability data under long-term conditions. Extrapolation of real-time data may be performed to extend the shelf-life [4]. As discussed and presented above, whether the data should be transformed for linear or non-linear regression analysis depends on the nature of the degradation kinetics. To test the influence of the regression model on the calculated shelf-life, the data were analyzed using linear and non-linear regression (Table 4). Note that the calculations of shelf-life for the comparison were performed without considering the 95 one-sided confidence limit.

In the case of Pemetrexed, the calculated shelf-life with linear regression was higher than that calculated with non-linear regression for all reduced models. However, the difference between the shelf-life calculated with 24-month data and that with 12-month data was smaller in the case of non-linear regression analysis. In addition, the prediction of the shelf-life with linear regression for Pemetrexed from 12-month data led to a large error. This was to be expected, as the degradation kinetics of the product were not linear, but the impurity increased exponentially after 12 months.

In the case of Sugammadex, the shelf-life could only be calculated using linear regression. Since the increase in degradation products was rapid up to 12 months and then slowed down, the non-linear curve did not approach the proposed shelf-life limit. If the data were analyzed with linear regression, the calculated shelf-life from 24-months data was higher compared to 12-months data. For Docetaxel, however, the shelf-life could only be calculated using linear regression, as the degradation kinetics were linear. The calculated shelf-life showed that the results were comparable if 12-month or 24-month data were used.

For an accurate prediction of shelf-life based on 12-month stability data, it is important to choose an appropriate regression model based on the type of impurity kinetics observed. For degradation kinetics where impurities initially increase slowly and then exponentially, a non-linear regression is recommended. Linear regression in such cases may lead to a longer predicted shelf-life, which can be misleading. In contrast, for degradation kinetics where impurities initially increase faster and then slow down, linear regression is preferable. If a non-linear regression model is used in these cases, the curve does not reach the specification limit. It is important to consider that with a linear regression, the predicted shelf-life may be shorter than it actually is, which is the worst-case scenario.

### 4.3. Adequacy of Matrixing Designs

To evaluate the level of comparability between the matrixing designs (M2–M29) and the full design (M1), we calculated the statistical parameter RMSE. A lower RMSE value indicates better similarity between the reduced design and the full design. From the calculated data (Table 3), it is evident that the RMSE values increased as the number of time points decreased. For non-linear regression analysis, the RMSE values remained below the set limit of 0.01%. However, for linear regression analysis, the RMSE values fell below the limit for the 5- or 6-point model.

Upon closer examination, we noted that for the non-linear regression analysis, the matrixing design M17 yielded the lowest RMSE value for Pemetrexed (Table S6), while the model M2 achieved the lowest RMSE for Sugammadex (Table S7). For Docetaxel, for which the data were analyzed with linear regression, the lower RMSE was calculated for model M20 (Table S8). The reduced models M17 and M20 are 5-time point models, while M2 is a 6-time point model. Nevertheless, Sugammadex can also be reduced to a 5-point model, for example M10, as its RMSE value is comparable to models M2–M4. This implies that a reduction in two time points per batch corresponds to approximately 30% and can be achieved for all the parenteral drug products examined.

The fact that the RMSE values remained below the set limit of 0.01% further confirmed the validity of reducing to either a 5-point or 6-point model.

Furthermore, if we check the reduced time points, we notice that for M20 and M10, the time points were reduced uniformly. For M17, on the other hand, the largest reduction in time points was before 12 months. This is understandable, as the shelf-life for degradation kinetics, such as Pemetrexed, is mainly determined by the second part of the stability study.

The obtained statistical parameter RMSE for all 28 reduced designs obtained with the corresponding regression model is shown graphically in Figure 2. From the RMSE distribution shown, the 4-point matrixing designs M26-M29 were mostly outliers. However, the other matrixing designs (M2–M25) were distributed within a certain variability, indicating comparable results with the full design (M1).

Comparing our results with the study conducted by DeWoody and Raghavarao on solid dosage forms [11], similarities can be found in the conclusions regarding the suitability of matrixing designs. In their study, they determined that the optimal matrixing design, by using different statistical parameters, was M5 (6-time point model) or design M11 (5-time point model). This is similar to our conclusions, where we suggest that the stability study can be reduced for two time points for each batch, leading to a 5-time point model.

## 5. Conclusions

The aim of our work was to investigate the applicability of matrixing designs for parenteral pharmaceutical dosage forms. While the existing literature focuses on the application to solid dosage forms, this study stands out due to the fact that it is the first to focus specifically on parenteral pharmaceutical dosage forms. We investigated three parenteral drug products with different degradation kinetics. The results demonstrate that a reduction of two time points for each batch, corresponding to a reduction of approximately 30%, is possible when using matrixing designs. Whether the degradation product shows a linear or non-linear increase does not impact the feasibility of using matrixing designs. Instead, proper evaluation of the stability data is important to ensure an accurate assessment. The nature of the degradation kinetics should determine whether a linear or non-linear regression analysis is appropriate for shelf-life determination, which can also be determined from the knowledge of the product from early stages of development and sufficient supporting data. By selecting the matrixing approach, the total number of samples required in a stability study can be sufficient to determine the stability characteristics of the drug product and estimate the shelf-life of the drug product with an acceptable degree of precision.

In summary, this study provides valuable insight into the applicability of matrixing designs to parenteral pharmaceutical dosage forms. With careful evaluation of stability data and appropriate study design, matrixing designs offer an effective strategy for updating stability testing in the pharmaceutical industry. Companies can achieve significant cost and time savings while maintaining the required level of precision and reliability in determining shelf-life. Further research in this area can improve our understanding and support the application of matrixing designs to other types of liquid dosage forms.

## Figures and Tables

**Table 1:** Full stability design of tested products for the proposed shelf-life of two years.

	Pemetrexed	Sugammadex	Docetaxel
Number of batches	3	3	3
Number of filling volumes	3	2	3
Number of orientations	2	2	2
Sampling time (months):			
Long-term testing	0, 3, 6, 9, 12, 18, 24	0, 3, 6, 9, 12, 18, 24	0, 3, 6, 9, 12, 18, 24
Accelerated testing	0, 3, 6	0, 3, 6	0, 3, 6
Intermediate testing	0, 6, 9, 12	0, 6, 9, 12	0, 6, 9, 12
Total number of samples tested	252	168	252

**Table 2:** List of the matrixing designs.

Design	Batch 1	Batch 2	Batch 3	No. of Time Points
M1 (full)	0, 3, 6, 9, 12, 18, 24	0, 3, 6, 9, 12, 18, 24	0, 3, 6, 9, 12, 18, 24	7
M2	0, 3, 6, 9, 12, 24	0, 3, 6, 12, 18, 24	0, 3, 9, 12, 18, 24	6
M3	0, 3, 6, 9, 12, 24	0, 3, 6, 12, 18, 24	0, 6, 9, 12, 18, 24	6
M4	0, 3, 6, 9, 12, 24	0, 3, 9, 12, 18, 24	0, 6, 9, 12, 18, 24	6
M5	0, 3, 6, 12, 18, 24	0, 3, 9, 12, 18, 24	0, 6, 9, 12, 18, 24	6
M6	0, 3, 6, 12, 24	0, 3, 9, 12, 24	0, 3, 12, 18, 24	5
M7	0, 3, 6, 12, 24	0, 3, 9, 12, 24	0, 6, 9, 12, 24	5
M8	0, 3, 6, 12, 24	0, 3, 9, 12, 24	0, 6, 12, 18, 24	5
M9	0, 3, 6, 12, 24	0, 3, 9, 12, 24	0, 9, 12, 18, 24	5
M10	0, 3, 6, 12, 24	0, 3, 12, 18, 24	0, 6, 9, 12, 24	5
M11	0, 3, 6, 12, 24	0, 3, 12, 18, 24	0, 6, 12, 18, 24	5
M12	0, 3, 6, 12, 24	0, 3, 12, 18, 24	0, 9, 12, 18, 24	5
M13	0, 3, 6, 12, 24	0, 6, 9, 12, 24	0, 6, 12, 18, 24	5
M14	0, 3, 6, 12, 24	0, 6, 9, 12, 24	0, 9, 12, 18, 24	5
M15	0, 3, 6, 12, 24	0, 6, 12, 18, 24	0, 9, 12, 18, 24	5
M16	0, 3, 9, 12, 24	0, 3, 12, 18, 24	0, 6, 9, 12, 24	5
M17	0, 3, 9, 12, 24	0, 3, 12, 18, 24	0, 6, 12, 18, 24	5
M18	0, 3, 9, 12, 24	0, 3, 12, 18, 24	0, 9, 12, 18, 24	5
M19	0, 3, 9, 12, 24	0, 6, 9, 12, 24	0, 6, 12, 18, 24	5
M20	0, 3, 9, 12, 24	0, 6, 9, 12, 24	0, 9, 12, 18, 24	5
M21	0, 3, 9, 12, 24	0, 6, 12, 18, 24	0, 9, 12, 18, 24	5
M22	0, 3, 12, 18, 24	0, 6, 9, 12, 24	0, 6, 12, 18, 24	5
M23	0, 3, 12, 18, 24	0, 6, 9, 12, 24	0, 9, 12, 18, 24	5
M24	0, 3, 12, 18, 24	0, 6, 12, 18, 24	0, 9, 12, 18, 24	5
M25	0, 6, 9, 12, 24	0, 6, 12, 18, 24	0, 9, 12, 18, 24	5
M26	0, 3, 12, 24	0, 6, 12, 24	0, 9, 12, 24	4
M27	0, 3, 12, 24	0, 6, 12, 24	0, 12, 18, 24	4
M28	0, 3, 12, 24	0, 9, 12, 24	0, 12, 18, 24	4
M29	0, 6, 12, 24	0, 9, 12, 24	0, 12, 18, 24	4

**Table 3:** Regression analysis for Pemetrexed, Sugammadex, and Docetaxel. With the exception of the full model (M1), the statistical parameters are presented as average values of all matrixing models with the same number of the time points.

		Non-Linear Regression			Linear Regression		
	Design (Time Points)	S, % ^1,4^	R^2^, % ^2^	RMSE, % ^3^	S, % ^1,4^	R^2^, % ^2^	RMSE, % ^3^
Pemetrexed	M1 (7)	0.03502	98.8	-	0.07655	94.3	-
	M2–M5 (6)	0.03633	98.9	0.00140	0.08029	94.2	0.00533
	M6–M25 (5)	0.03546	99.0	0.00411	0.08505	94.1	0.00970
	M26–M29 (4)	0.03418	99.2	0.00886	0.09165	94.1	0.01550
Sugammadex	M1 (7)	0.01814	97.6	-	0.03823	88.9	-
	M2–M5 (6)	0.01885	97.6	0.00133	0.04033	88.8	0.00281
	M6–M25 (5)	0.01975	97.7	0.00298	0.04236	89.0	0.00710
	M26–M29 (4)	0.02059	97.9	0.00551	0.04430	89.9	0.01373
Docetaxel	M1 (7)	-	-	-	0.07160	96.8	-
	M2–M5 (6)	-	-	-	0.07089	97.1	0.00326
	M6–M25 (5)	-	-	-	0.07039	97.4	0.00756
	M26–M29 (4)	-	-	-	0.06991	97.8	0.01435

^1^ Standard error of the regression (S), ^2^ coefficient of determination (R^2^), ^3^ root-mean-square error (RMSE). ^4^ Standard error of the regression (S) represents the whole uncertainty or variation, such as the batch-to-batch variation; the error of the analytical method; the uncertainty of the fitted model; and, in our study, also the error due to reduced matrixing designs.

**Table 4:** Calculated shelf-life with 24-month data vs. 12-month data using a linear and a non-linear regression model. Shelf-life (in months) is presented as an average value.

	Linear Regression						Non-Linear Regression	
Design	Pemetrexed		Sugammadex		Docetaxel		Pemetrexed	
(Time Points)	24 m	12 m	24 m	12 m	24 m	12 m	24 m	12 m
M1 (7)	33.4	51.2	38.9	27.1	28.4	28.8	27.7	31.8
M2–M5 (6)	33.2	51.4	38.8	27.1	28.3	28.8	27.7	32.4
M6–M25 (5)	33.1	51.2	38.8	27.2	28.3	27.9	27.7	32.5
M26–M29 (4)	32.9	51.5	38.8	27.4	28.2	27.5	27.7	31.8

## Data Availability

Data are contained within the article and Supplementary Materials.

## Supplementary Materials

The following supporting information can be downloaded at: https://www.mdpi.com/article/10.3390/pharmaceutics16091117/s1, Table S1: Chromatographic conditions of analytical methods for impurities; Table S2: Stability data (impurities) for three filling volumes of Pemetrexed; Table S3: Stability data (impurities) for two filling volumes of Sugammadex; Table S4: Stability data (impurities) for three filling volumes of Docetaxel; Table S5: Stability data (impurities) of less stable filling volumes used for statistical evaluation; Table S6: Linear and non-linear regression analysis for Pemetrexed; Table S7: Linear and non-linear regression analysis for Sugammadex; Table S8: Linear regression analysis for Docetaxel; Table S9: Calculated shelf-life with 24 months data vs. 12 months data using a linear and non-linear regression model; Figure S1: Structure of examined compounds; Figure S2: Structure of monitored degradation products.
