# Supplementary material for: Matrixing Designs for Shelf-Life Determination of Parenteral Drug Product: A Comparative Analysis of Full and Reduced Stability Testing Design
Source: Pharmaceutics. 2024 Aug 24;16(9):1117. doi: 10.3390/pharmaceutics16091117 (PMC11435299; doi:10.3390/pharmaceutics16091117)
Supplement: Supplementary file 1 [file pharmaceutics-16-01117-s001.zip › pharmaceutics-3118075-supplementary.pdf]

Article

Matrixing designs for shelf-life determination of parenteral drug product: A comparative analysis of full and reduced stability testing design

Lara Pavčnik [1,2], Igor Locatelli [2], Tina Trdan Lušin [1,2] and Robert Roškar [2] *

[1] SANDOZ Development Center Slovenia, Lek Pharmaceuticals d.d., Verovškova 57, SI-1526 Ljubljana, SLOVENIA, lara.pavcnik@sandoz.com (L.P.); tina.trdan_lusin@sandoz.com (T.T.L.)
[2] Department of Biopharmaceutics and Pharmacokinetics, Faculty of Pharmacy, University of Ljubljana, SI-1000 Ljubljana, Slovenia; igor.locatelli@ffa.uni-lj.si (I.L.) robert.roskar@ffa.uni-lj.si (R.R.)
* Correspondence: robert.roskar@ffa.uni-lj.si

List of Tables:

Table S1: Chromatographic conditions of analytical methods for impurities. 2
Table S2: Stability data (impurities) for three filling volumes of Pemetrexed. 3
Table S3: Stability data (impurities) for two filling volumes of Sugammadex. 4
Table S4: Stability data (impurities) for three filling volumes of Docetaxel. 5
Table S5: Stability data (impurities) of less stable filling volumes used for statistical evaluation. 6
Table S6: Linear and non-linear regression analysis for Pemetrexed. 7
Table S7: Linear and non-linear regression analysis for Sugammadex. 8
Table S8: Linear regression analysis for Docetaxel. 9
Table S9: Calculated shelf-life with 24 months data vs. 12 months data using a linear and non-linear regression model. 10

List of Figures:

Figure S1: Structure of examined compounds. 11
Figure S2: Structure of monitored degradation products. 11

Table S1: Chromatographic conditions of analytical methods for impurities.

<table>
<tr><td></td><td>Pemetrexed</td><td>Sugammadex</td><td>Docetaxel</td></tr>
<tr><td></td><td>HPLC[1]</td><td>UPLC[2]</td><td>HPLC[1]</td></tr>
<tr><td>Column</td><td>Poroshell 120 SB-C18
2.7 µm, 100 × 3.0 mm</td><td>Acquity UPLC Protein BEH C4
1.7 µm, 150 × 2.1 mm</td><td>Zorbax SB-C18
1.8 µm, 50 × 4.6 mm</td></tr>
<tr><td>Mobile phase</td><td>A: 0.02M KH_2PO_4, pH = 3.0
B: Acetonitrile</td><td>A: 0.01M NaH_2PO_4, pH = 2.5
B: Acetonitrile</td><td>A: Purified water: acetonitrile = 60 : 40 (V/V)
B: Acetonitrile</td></tr>
<tr><td>Solvent</td><td>Purified water</td><td>Purified water: acetonitrile = 92 : 8 (V/V)</td><td>Purified water: acetonitrile = 60 : 40 (V/V)</td></tr>
<tr><td>Column temperature</td><td>35°C</td><td>46°C</td><td>35°C</td></tr>
<tr><td>Flow rate</td><td>1.4 ml/min</td><td>0.33 ml/min</td><td>1.8 ml/min</td></tr>
<tr><td>Gradient</td>
<td><table>
<tr><td>Time (min)</td><td>% A</td><td>% B</td></tr>
<tr><td>0</td><td>91</td><td>9</td></tr>
<tr><td>6.8</td><td>91</td><td>9</td></tr>
<tr><td>13.6</td><td>80</td><td>20</td></tr>
<tr><td>15.6</td><td>80</td><td>20</td></tr>
<tr><td>15.8</td><td>91</td><td>9</td></tr>
<tr><td>18.0</td><td>91</td><td>9</td></tr>
</table></td>
<td><table>
<tr><td>Time (min)</td><td>% A</td><td>% B</td></tr>
<tr><td>0</td><td>90</td><td>10</td></tr>
<tr><td>1</td><td>90</td><td>10</td></tr>
<tr><td>2</td><td>86</td><td>14</td></tr>
<tr><td>8.5</td><td>81.5</td><td>18.5</td></tr>
<tr><td>18.5</td><td>81.5</td><td>18.5</td></tr>
<tr><td>24.5</td><td>75.5</td><td>24.5</td></tr>
<tr><td>31</td><td>55</td><td>45</td></tr>
<tr><td>34</td><td>55</td><td>45</td></tr>
<tr><td>34.5</td><td>20</td><td>80</td></tr>
<tr><td>36</td><td>20</td><td>80</td></tr>
<tr><td>37</td><td>90</td><td>10</td></tr>
</table></td>
<td><table>
<tr><td>Time (min)</td><td>% A</td><td>% B</td></tr>
<tr><td>0</td><td>92</td><td>8</td></tr>
<tr><td>3.55</td><td>92</td><td>8</td></tr>
<tr><td>8.00</td><td>54.5</td><td>45.5</td></tr>
<tr><td>8.60</td><td>0</td><td>100</td></tr>
<tr><td>16.89</td><td>0</td><td>100</td></tr>
<tr><td>17.78</td><td>92</td><td>8</td></tr>
<tr><td>20.00</td><td>92</td><td>8</td></tr>
</table></td></tr>
<tr><td>Wavelength</td><td>228 nm</td><td>205 nm</td><td>230 nm</td></tr>
<tr><td>Injection volume</td><td>5 µl</td><td>2 µl</td><td>8 µl</td></tr>
<tr><td>Sampler temperature</td><td>5°C</td><td>5°C</td><td>21°C</td></tr>
</table>

[1] High-performance liquid chromatography (HPLC), [2] Ultra-performance liquid chromatography (UPLC)

Table S2: Stability data (impurities) for three filling volumes of Pemetrexed.

		Time point, months	Pemetrexed 100mg/4ml	Pemetrexed 500mg/20ml	Pemetrexed 1000mg/40ml
Batch	Orientation		IMP1[1], %	IMP1[1], %	IMP1[1], %
1	Inverted	0	0.00	0.00	0.00
1	Inverted	3	0.06	0.04	0.03
1	Inverted	6	0.12	0.05	0.03
1	Inverted	9	0.22	0.04	0.03
1	Inverted	12	0.34	0.09	0.05
1	Inverted	18	0.61	0.10	0.08
1	Inverted	24	1.00	0.15	0.08
2	Inverted	0	0.00	0.00	0.00
2	Inverted	3	0.09	0.00	0.00
2	Inverted	6	0.12	0.06	0.05
2	Inverted	9	0.21	0.05	0.04
2	Inverted	12	0.28	0.08	0.07
2	Inverted	18	0.68	0.10	0.09
2	Inverted	24	0.97	0.14	0.06
3	Inverted	0	0.00	0.00	0.00
3	Inverted	3	0.04	0.03	0.00
3	Inverted	6	0.11	0.05	0.03
3	Inverted	9	0.19	0.05	0.04
3	Inverted	12	0.27	0.08	0.05
3	Inverted	18	0.58	0.10	0.07
3	Inverted	24	0.96	0.14	0.06
1	Upright	0	0.00	0.00	0.00
1	Upright	3	0.05	0.03	0.00
1	Upright	6	0.10	0.04	0.03
1	Upright	9	0.21	0.06	0.03
1	Upright	12	0.30	0.08	0.05
1	Upright	18	0.52	0.11	0.07
1	Upright	24	0.91	0.12	0.07
2	Upright	0	0.00	0.00	0.00
2	Upright	3	0.04	0.00	0.06
2	Upright	6	0.11	0.03	0.04
2	Upright	9	0.16	0.04	0.05
2	Upright	12	0.27	0.07	0.06
2	Upright	18	0.55	0.10	0.08
2	Upright	24	0.88	0.12	0.09
3	Upright	0	0.00	0.00	0.00
3	Upright	3	0.04	0.03	0.03
3	Upright	6	0.10	0.05	0.03
3	Upright	9	0.21	0.05	0.04
3	Upright	12	0.23	0.08	0.05
3	Upright	18	0.51	0.11	0.06
3	Upright	24	0.87	0.13	0.06

[1] Oxidative degradation product pemetrexed S-dimer (IMP1).

Table S3: Stability data (impurities) for two filling volumes of Sugammadex.

		Time point, months	Sugammadex 200mg/2ml	Sugammadex 500mg/5ml
Batch	Orientation		IMP1[1], %	IMP1[1], %
1	Horizontal	0	0.25	0.23
1	Horizontal	3	0.33	0.31
1	Horizontal	6	0.38	0.36
1	Horizontal	9	0.44	0.42
1	Horizontal	12	0.48	0.46
1	Horizontal	18	0.53	0.52
1	Horizontal	24	0.57	0.56
2	Horizontal	0	0.18	0.14
2	Horizontal	3	0.32	0.21
2	Horizontal	6	0.38	0.27
2	Horizontal	9	0.42	0.34
2	Horizontal	12	0.45	0.39
2	Horizontal	18	0.52	0.45
2	Horizontal	24	0.55	0.49
3	Horizontal	0	0.21	0.19
3	Horizontal	3	0.33	0.29
3	Horizontal	6	0.39	0.37
3	Horizontal	9	0.44	0.43
3	Horizontal	12	0.48	0.47
3	Horizontal	18	0.53	0.51
3	Horizontal	24	0.57	0.55
1	Upright	0	0.25	0.23
1	Upright	3	0.33	0.31
1	Upright	6	0.38	0.36
1	Upright	9	0.44	0.42
1	Upright	12	0.48	0.45
1	Upright	18	0.52	0.50
1	Upright	24	0.56	0.55
2	Upright	0	0.18	0.14
2	Upright	3	0.31	0.21
2	Upright	6	0.37	0.27
2	Upright	9	0.43	0.34
2	Upright	12	0.45	0.39
2	Upright	18	0.52	0.44
2	Upright	24	0.54	0.48
3	Upright	0	0.21	0.19
3	Upright	3	0.32	0.29
3	Upright	6	0.39	0.37
3	Upright	9	0.43	0.42
3	Upright	12	0.47	0.45
3	Upright	18	0.53	0.51
3	Upright	24	0.57	0.55

[1] Oxidative degradation product mono-S-oxo-sugammadex (IMP1).

Table S4: Stability data (impurities) for three filling volumes of Docetaxel.

Batch	Orientation	Time point, months	Docetaxel 20mg/2ml IMP1[1], %	Docetaxel 80mg/8ml IMP1[1], %	Docetaxel 160mg/16ml IMP1[1], %
1	Inverted	0	0.15	0.15	0.16
1	Inverted	3	0.24	0.22	0.28
1	Inverted	6	0.36	0.35	0.35
1	Inverted	9	0.46	0.42	0.44
1	Inverted	12	0.82	0.70	0.68
1	Inverted	18	1.04	1.00	0.76
1	Inverted	24	1.40	1.28	1.00
2	Inverted	0	0.13	0.12	0.14
2	Inverted	3	0.25	0.22	0.34
2	Inverted	6	0.38	0.35	0.50
2	Inverted	9	0.55	0.50	0.53
2	Inverted	12	0.80	0.77	0.77
2	Inverted	18	1.03	0.95	0.87
2	Inverted	24	1.33	1.20	1.00
3	Inverted	0	0.12	0.11	0.10
3	Inverted	3	0.37	0.25	0.30
3	Inverted	6	0.51	0.41	0.38
3	Inverted	9	0.60	0.55	0.45
3	Inverted	12	0.85	0.80	0.51
3	Inverted	18	1.05	0.95	0.60
3	Inverted	24	1.35	1.23	0.69
1	Upright	0	0.15	0.15	0.16
1	Upright	3	0.25	0.23	0.25
1	Upright	6	0.32	0.34	0.29
1	Upright	9	0.39	0.41	0.35
1	Upright	12	0.66	0.69	0.59
1	Upright	18	0.91	0.92	0.64
1	Upright	24	1.25	1.23	0.92
2	Upright	0	0.13	0.11	0.14
2	Upright	3	0.22	0.21	0.29
2	Upright	6	0.35	0.40	0.52
2	Upright	9	0.47	0.49	0.55
2	Upright	12	0.66	0.82	0.73
2	Upright	18	0.95	1.00	0.80
2	Upright	24	1.15	1.19	0.85
3	Upright	0	0.12	0.11	0.10
3	Upright	3	0.23	0.22	0.31
3	Upright	6	0.35	0.39	0.43
3	Upright	9	0.48	0.45	0.50
3	Upright	12	0.65	0.66	0.58
3	Upright	18	0.95	0.93	0.65
3	Upright	24	1.25	1.20	0.75

[1] Oxidative degradation product 6-oxodocetaxel (IMP1).

Table S5: Stability data (impurities) of less stable filling volumes used for statistical evaluation.

Batch	Orientation*	Time point, months	Pemetrexed 100mg/4ml IMP1[1], %	Sugammadex 200mg/2ml IMP1[2], %	Docetaxel 20mg/2ml IMP1[3], %
1	Inverted	0	0.00	0.25	0.15
1	Inverted	3	0.06	0.33	0.24
1	Inverted	6	0.12	0.38	0.36
1	Inverted	9	0.22	0.44	0.46
1	Inverted	12	0.34	0.48	0.82
1	Inverted	18	0.61	0.53	1.04
1	Inverted	24	1.00	0.57	1.40
2	Inverted	0	0.00	0.18	0.13
2	Inverted	3	0.09	0.32	0.25
2	Inverted	6	0.12	0.38	0.38
2	Inverted	9	0.21	0.42	0.55
2	Inverted	12	0.28	0.45	0.80
2	Inverted	18	0.68	0.52	1.03
2	Inverted	24	0.97	0.55	1.33
3	Inverted	0	0.00	0.21	0.12
3	Inverted	3	0.04	0.33	0.37
3	Inverted	6	0.11	0.39	0.51
3	Inverted	9	0.19	0.44	0.60
3	Inverted	12	0.27	0.48	0.85
3	Inverted	18	0.58	0.53	1.05
3	Inverted	24	0.96	0.57	1.35
1	Upright	0	0.00	0.25	0.15
1	Upright	3	0.05	0.33	0.25
1	Upright	6	0.10	0.38	0.32
1	Upright	9	0.21	0.44	0.39
1	Upright	12	0.30	0.48	0.66
1	Upright	18	0.52	0.52	0.91
1	Upright	24	0.91	0.56	1.25
2	Upright	0	0.00	0.18	0.13
2	Upright	3	0.04	0.31	0.22
2	Upright	6	0.11	0.37	0.35
2	Upright	9	0.16	0.43	0.47
2	Upright	12	0.27	0.45	0.66
2	Upright	18	0.55	0.52	0.95
2	Upright	24	0.88	0.54	1.15
3	Upright	0	0.00	0.21	0.12
3	Upright	3	0.04	0.32	0.23
3	Upright	6	0.10	0.39	0.35
3	Upright	9	0.21	0.43	0.48
3	Upright	12	0.23	0.47	0.65
3	Upright	18	0.51	0.53	0.95
3	Upright	24	0.87	0.57	1.25

* Product Sugammadex sodium was tested in horizontal and upright position.

[1] Oxidative degradation product pemetrexed S-dimer (IMP1), [2] oxidative degradation product mono-S-oxo-sugammadex (IMP1), [3] oxidative degradation product 6-oxodocetaxel (IMP1).

Table S6: Linear and non-linear regression analysis for Pemetrexed.

	Non-linear regression			Linear regression		
Design	S, %[1]	R^2, %[2]	RMSE, %[3]	S, %[1]	R^2, %[2]	RMSE, %[3]
M1	0.03502	98.8		0.07655	94.3	
M2	0.03578	98.9	0.00132	0.07859	94.5	0.00843
M3	0.03577	98.9	0.00134	0.07927	94.3	0.00611
M4	0.03630	98.8	0.00048	0.08131	94.0	0.00370
M5	0.03746	98.8	0.00230	0.08197	94.0	0.00309
M6	0.03236	99.2	0.00814	0.08421	94.4	0.01282
M7	0.03094	99.2	0.00412	0.08437	94.1	0.01250
M8	0.03238	99.2	0.00777	0.08520	94.2	0.00970
M9	0.03277	99.2	0.00625	0.08621	93.9	0.00818
M10	0.03704	99.0	0.00229	0.08287	94.6	0.01674
M11	0.03873	98.9	0.00287	0.08366	94.6	0.01502
M12	0.03891	98.8	0.00144	0.08478	94.3	0.01356
M13	0.03135	99.2	0.00751	0.08494	94.1	0.00672
M14	0.03175	99.2	0.00621	0.08590	93.9	0.00508
M15	0.03802	98.9	0.00197	0.08481	94.2	0.00963
M16	0.03741	98.9	0.00360	0.08338	94.4	0.01557
M17	0.03925	98.8	0.00034	0.08410	94.4	0.01424
M18	0.03936	98.8	0.00117	0.08519	94.1	0.01282
M19	0.03230	99.2	0.00518	0.08532	94.0	0.00560
M20	0.03258	99.1	0.00401	0.08626	93.7	0.00396
M21	0.03849	98.8	0.00091	0.08522	94.0	0.00877
M22	0.03347	99.1	0.00806	0.08611	94.0	0.00466
M23	0.03386	99.1	0.00652	0.08702	93.7	0.00328
M24	0.03985	98.8	0.00270	0.08593	94.0	0.00897
M25	0.03847	98.8	0.00121	0.08554	93.9	0.00613
M26	0.03269	99.3	0.00456	0.08909	94.4	0.01981
M27	0.03436	99.2	0.00950	0.09003	94.4	0.01820
M28	0.03484	99.2	0.01081	0.09334	93.8	0.01435
M29	0.03481	99.2	0.01058	0.09414	93.6	0.00963

[1] Standard error of the regression (S), [2] coefficient of determination (R^2), [3] root-mean-square error (RMSE).

Table S7: Linear and non-linear regression analysis for Sugammadex.

	Non-linear regression			Linear regression		
Design	S, %[1]	R^2, %[2]	RMSE, %[3]	S, %[1]	R^2, %[2]	RMSE, %[3]
M1	0.01814	97.6		0.03823	88.9	
M2	0.01897	97.6	0.00097	0.03990	89.2	0.00357
M3	0.01876	97.6	0.00120	0.04073	88.4	0.00213
M4	0.01880	97.6	0.00116	0.04106	88.1	0.00165
M5	0.01885	97.7	0.00200	0.03962	89.4	0.00387
M6	0.02019	97.7	0.00219	0.04074	90.1	0.00882
M7	0.01986	97.5	0.00312	0.04336	87.7	0.00570
M8	0.01994	97.7	0.00226	0.04196	89.2	0.00727
M9	0.01941	97.8	0.00296	0.04226	89.1	0.00703
M10	0.02042	97.5	0.00167	0.04242	88.9	0.00736
M11	0.02040	97.7	0.00185	0.04087	90.3	0.00917
M12	0.01986	97.8	0.00293	0.04117	90.1	0.00896
M13	0.01970	97.6	0.00271	0.04246	88.6	0.00604
M14	0.01912	97.8	0.00347	0.04277	88.4	0.00578
M15	0.01960	97.8	0.00346	0.04176	89.5	0.00770
M16	0.02049	97.5	0.00175	0.04369	88.2	0.00600
M17	0.02050	97.6	0.00171	0.04222	89.6	0.00790
M18	0.01998	97.8	0.00264	0.04250	89.4	0.00786
M19	0.01977	97.6	0.00282	0.04369	87.9	0.00472
M20	0.01920	97.7	0.00341	0.04396	87.3	0.00467
M21	0.01970	97.7	0.00329	0.04304	88.7	0.00660
M22	0.01986	97.7	0.00334	0.04180	89.4	0.00733
M23	0.01921	97.8	0.00439	0.04208	89.3	0.00740
M24	0.01957	97.8	0.00503	0.04094	90.2	0.00955
M25	0.01817	98.0	0.00467	0.04341	88.2	0.00617
M26	0.02123	97.7	0.00401	0.04555	88.9	0.01220
M27	0.02116	97.9	0.00487	0.04327	90.6	0.01473
M28	0.02102	97.9	0.00579	0.04391	90.3	0.01426
M29	0.01893	98.2	0.00735	0.04447	89.7	0.01373

[1] Standard error of the regression (S), [2] coefficient of determination (R^2), [3] root-mean-square error (RMSE).

Table S8: Linear regression analysis for Docetaxel.

	Linear regression		
Design	S, %[1]	R^2, %[2]	RMSE, %[3]
M1	0.07160	96.8	
M2	0.07185	97.1	0.00206
M3	0.07191	97.0	0.00266
M4	0.07256	96.9	0.00192
M5	0.06722	97.4	0.00641
M6	0.06868	97.6	0.01011
M7	0.06968	97.3	0.00818
M8	0.06897	97.5	0.00935
M9	0.06740	97.6	0.00721
M10	0.06855	97.6	0.01052
M11	0.06778	97.7	0.01166
M12	0.06630	97.8	0.00940
M13	0.06961	97.4	0.00826
M14	0.06800	97.5	0.00609
M15	0.06697	97.7	0.00823
M16	0.07517	97.0	0.00623
M17	0.07454	97.2	0.00753
M18	0.07306	97.3	0.00514
M19	0.07600	96.9	0.00392
M20	0.07440	97.0	0.00187
M21	0.07359	97.1	0.00399
M22	0.06921	97.5	0.01108
M23	0.06779	97.5	0.00869
M24	0.06653	97.7	0.01165
M25	0.07561	96.9	0.00218
M26	0.06935	97.8	0.01410
M27	0.06825	97.9	0.01591
M28	0.06923	97.8	0.01586
M29	0.07282	97.5	0.01151

[1] Standard error of the regression (S), [2] coefficient of determination (R^2), [3] root-mean-square error (RMSE).

Table S9: Calculated shelf-life with 24 months data vs. 12 months data using a linear and non-linear regression model.

	Linear regression						Non- linear regression	
Design	Pemetrexed		Sugammadex		Docetaxel		Pemetrexed	
	24m	12m	24m	12m	24m	12m	24m	12m
M1	33.4	51.2	38.9	27.1	28.4	28.8	27.7	31.8
M2	33.2	50.7	38.9	27.2	28.3	30.3	27.8	33.0
M3	33.1	51.2	38.9	26.9	28.3	28.4	27.7	32.5
M4	33.2	51.6	38.8	26.9	28.3	28.6	27.7	32.4
M5	33.4	52.0	38.7	27.2	28.3	27.7	27.7	31.6
M6	33.1	51.4	39.1	27.8	28.2	27.7	27.7	31.3
M7	32.7	52.0	39.3	27.2	28.2	27.7	27.7	31.6
M8	33.1	51.9	39.1	27.4	28.2	27.6	27.7	31.2
M9	33.2	51.9	38.8	27.3	28.2	27.8	27.7	32.6
M10	32.7	51.5	39.2	27.2	28.2	27.4	27.7	31.9
M11	33.0	51.4	39.0	27.5	28.2	27.1	27.7	31.4
M12	33.1	51.3	38.8	27.4	28.2	27.4	27.7	33.4
M13	33.0	51.9	39.1	27.2	28.2	27.8	27.7	30.7
M14	33.1	51.8	38.8	27.1	28.2	27.9	27.7	31.7
M15	33.0	51.4	38.8	27.2	28.2	27.6	27.7	32.1
M16	32.8	51.1	39.0	27.0	28.3	28.2	27.7	34.1
M17	33.1	50.9	38.8	27.3	28.3	28.1	27.7	34.0
M18	33.2	50.9	38.5	27.2	28.3	28.3	27.8	39.1
M19	33.1	51.5	38.9	27.0	28.3	28.6	27.8	31.9
M20	33.2	51.5	38.7	26.9	28.3	28.7	27.8	33.7
M21	33.1	51.0	38.6	26.9	28.3	28.4	27.8	34.9
M22	33.3	45.2	38.7	27.3	28.3	27.6	27.7	26.8
M23	33.4	51.8	38.5	27.2	28.3	27.8	27.7	32.3
M24	33.3	51.3	38.4	27.3	28.3	27.4	27.7	33.1
M25	33.0	51.3	38.5	26.5	28.3	28.6	27.7	33.1
M26	32.6	51.3	39.2	27.3	28.2	27.4	27.7	33.1
M27	33.0	51.1	38.8	27.7	28.2	27.2	27.7	32.4
M28	33.1	51.6	38.6	27.6	28.2	27.5	27.7	31.3
M29	33.0	51.9	38.5	27.1	28.2	27.8	27.7	30.2

Pemetrexed	Sugammadex	Docetaxel
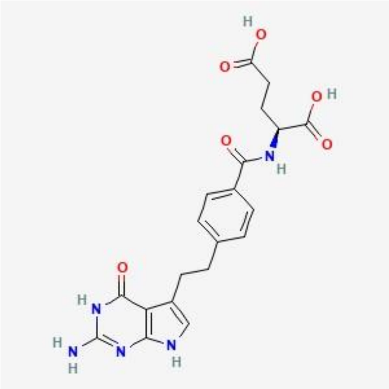	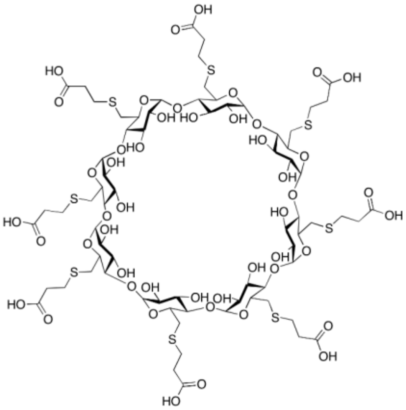	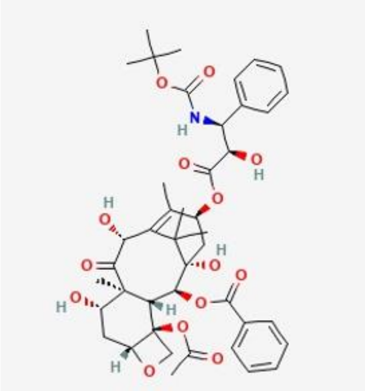

Figure S1: Structure of examined compounds.

pemetrexed S-dimer	mono-S-oxo-sugammadex	6-oxodocetaxel
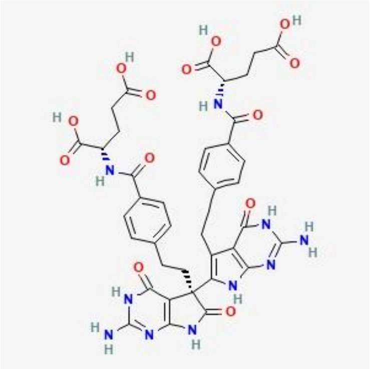	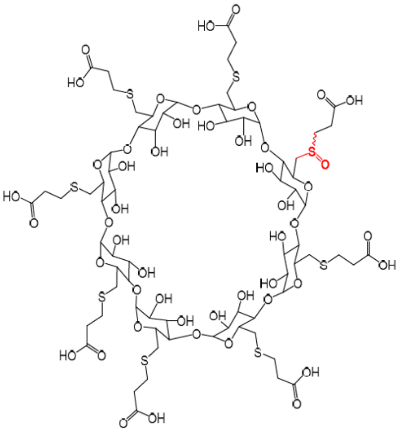	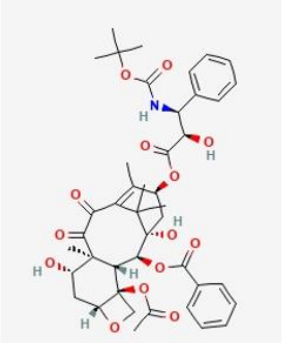

Figure S2: Structure of monitored degradation products.
